# Supplementary material for: Investigation of the inhibitory potential of secondary metabolites isolated from Fernandoa adenophylla against Beta-glucuronidase via molecular docking and molecular dynamics simulation studies
Source: PLoS One. 2025 May 23;20(5):e0324100. doi: 10.1371/journal.pone.0324100 (PMC12101693; doi:10.1371/journal.pone.0324100)
Supplement: S1 File — (DOC) [file pone.0324100.s001.doc]

**Supplementary file**

**Investigation of the inhibitory potential of secondary metabolites isolated from *Fernandoa adenophylla* against Beta-glucuronidase *via* molecular docking and molecular dynamics simulation studies**

Abdur Rauf^*1^, Rahaf Ajaj^2^, Zuneera Akram^3^, Majid Khan^4^, Abdul Wadood^5^, Maryam Zulfat^5^, Zafar Ali Shah^6^, Walaa F. Alsanie^7,8^, Abdulhakeem S. Alamri^7,8^, Majid Alhomrani^7,8^, Humaira Hussain^4^, Dorota Formanowicz^9^

1 Department of Chemistry, University of Swabi, Swabi, Anbar, 23430, Khyber Pakhtunkhwa (K.P.), Pakistan; mashaljcs@yahoo.com (A.R.);

2 Department of Environmental and Public Health, College of Health Sciences, Abu Dhabi University, Abu Dhabi, United Arab Emirates

3 Department of Pharmacology, Faculty of Pharmaceutical Sciences, University of Karachi, Karachi, Pakistan; dr.zunaira@baqai.edu.pk (Z.A.);

4 Department of Biochemistry, Abbottabad University of Science & Technology, Abbottabad, 22010, Pakistan; majidk166@yahoo.com (M.K.); humairahussainbch@gmail.com (H.H.);

5 Department of Biochemistry, Abdul Wali Khan University Mardan, Mardan, KPK, Pakistan; awadood@awkum.edu.pk (A.W.); maryamzulfat54@gmail.com (M.Z.);

6 Department of Agricultural Chemistry and Biochemistry, The University of Agriculture, Peshawar, Peshawar, Pakistan; zafarali@aup.edu.pk (Z.A.S);

7 Department of Clinical Laboratory Sciences, The Faculty of Applied Medical Sciences, Taif University, Taif, Saudi Arabia; w.alsanie@tu.edu.sa (W.F.A.), a.alamri@tu.edu.sa (A.S.A.), m.alhomrani@tu.edu.sa (M.A.);

8 Research Center for Health Sciences, Deanship of Graduate Studies and Scientific Research, Taif University, Taif 26432, Saudi Arabia;

9 Chair and Department of Medical Chemistry and Laboratory Medicine, Poznan University of Medical Sciences, Rokietnicka 8, 60-806 Poznan, Poland; doforman@ump.edu.pl (D.F.)

* Corresponding author: Abdur Rauf- [mashaljcs@yahoo.com](mailto:mashaljcs@yahoo.com); Rahaf Ajaj; rahaf.ajaj@adu.ac.ae

**Structure elucidation of isolated compounds (1-5)**

**Lapachol (1)**

It was a yellow crystalline compound, melting point 141-142 °C, molecular formula; C_15_H_14_O_3_, (m. wt 242.09) calculated from +TOF MS spectra m/z 241.0886 (M^+^-H).

**Table** 1. ^1^H NMR (500 MHz), ^13^C NMR (100 MHz), and HMBC correlations of lapachol in CDCl_3_

| **C. No.** | **^13^C NMR (δ, ppm)** | **Multiplicity** | **^1^H NMR (δ, ppm) (J in Hz)** | **HMBC Correlation** |
| --- | --- | --- | --- | --- |
| 1 | 181.1 | C | - | - |
| 2 | 152.6 | C | - | - |
| 3 | 123.5 | C | - | - |
| 4 | 184.4 | C | - | - |
| 5 | 126.1 | CH | 8.02 (1H, dd, J = 9.0, 1.5) | C-1, C-6 |
| 6 | 134.9 | CH | 7.69 (1H, dt, J = 9.0, 1.0) | C-5, C-8 |
| 7 | 132.9 | CH | 7.59 (1H, dt, J = 9.0, 1.5) | C-6, C-8, C-9 |
| 8 | 126.8 | CH | 8.12 (1H, dd, J = 9.0, 1.0) | C-4, C-7, C-9 |
| 9 | 129.4 | C | - | - |
| 10 | 129.4 | C | - | - |
| 1' | 22.6 | CH2 | 3.29 (2H, d, J = 7.5) | C-3, C-4, C-3', C-4', C-5' |
| 2' | 119.6 | CH | 5.20 (1H, t, J = 7.5) | C-4', C-5' |
| 3' | 133.8 | C | - | - |
| 4' | 17.8 | CH3 | 1.90 (3H, s) | C-2', C-3', C-5' |
| 5' | 25.7 | CH3 | 1.70 (3H, s) | C-2', C-3', C-4' |

**α-Lapachone (2)**

yellow crystalline compound, melting point 117 °C, molecular formula; C_15_H_14_O_3_ (m. wt. 242.09) deduced from EI^+^ m/z (242.0).

**Table** **2.** ^1^H NMR (600 MHz), ^13^C NMR (100 MHz) and HMBC correlations of α- lapachone in CDCl_3_

| **C. No.** | **^13^C NMR (δ, ppm)** | **Multiplicity** | **^1^H NMR (δ, ppm) (J in Hz)** | **HMBC Correlation** |
| --- | --- | --- | --- | --- |
| 1 | 180.0 | C | - | - |
| 2 | 154.6 | C | - | - |
| 3 | 120.1 | C | - | - |
| 4 | 184.4 | C | - | - |
| 5 | 125.9 | CH | 8.05 (1H, d, J = 6.6) | C-4, C-9 |
| 6 | 132.9 | CH | 7.67 (1H, dd, J = 6.6, 7.2) | C-7, C-8, C-10 |
| 7 | 132.9 | CH | 7.64 (1H, dd, J = 7.2, 7.8) | C-5, C-10 |
| 8 | 126.3 | CH | 8.07 (1H, d, J = 7.8) | C-1, C-10 |
| 9 | 132.0 | C | - | - |
| 10 | 131.1 | C | - | - |
| 11 | 16.7 | CH_2_ | 2.59 (2H, t, J = 6.0) | C-2, C-3, C-12, C-13 |
| 12 | 31.4 | CH_2_ | 1.80 (2H, t, J = 6.0) | C-3, C-11, C-14, C-15 |
| 13 | 78.7 | C | - | - |
| 14 | 26.9 | CH_3_ | 1.41 (3H, s) | C-15 |
| 15 | 26.5 | CH_3_ | 1.41 (3H, s) | C-12, C-13, C-14 |

**Peshawaraquinone (3)**

Yellow crystalline solid, m.p.: 250-252 °C. HR-ESIMS: 480.1594 [M-H] + (Calculated for C_37_H_39_N_2_O_7_= 480.16). UV (CHCl_3_) λ_max_ (log ε): 250 (6.99), 270 (5.5), 280 (5.61) nm. IR ῡ: 3441.01 (O-H, st.),1760.80, 1680, 1680.1, 1680.2 (C=O, carbonyl st.) cm-1. ^1^H and ^13^C-NMR:

**Table 3.** 1H NMR and 13C NMR spectroscopic data for peshawaraquinone in CDCl3

| **C. No.** | **^13^C (δ)** | **^1^H (δ) (*J* in Hz)** | **C. No** | | **13C** | **^1^H (δ) (*JHH* Hz)** |
| --- | --- | --- | --- | --- | --- | --- |
| 1 | 184.4 |  |  | 1′ | 193.5 |  |
| 2 | 120.7 |  |  | 2′ | 73.4 |  |
| 3 | 154.9 |  |  | 3′ | 203.6 |  |
| 4 | 179.3 |  |  | 4′ | 85.8 |  |
| 5 | 126.7 | 8.12 (dd, 8.4, 1.6) | 5′ | 124.8 |  | 7.92 (dd, 8.0, 1.5) |
| 6 | 133.6 | 7.74 (m) | 6′ | 135.5 |  | 7.74 (m) |
| 7 | 134.3 | 7.74, (m) | 7′ | 129.1 |  | 7.51 (m) |
| 8 | 126.7 | 8.16 (dd, 8.4, 1.6) | 8′ | 127.6 |  | 8.04 (dd, 7.6, 1.5) |
| 9 | 131.9 |  |  | 9′ | 129.7 |  |
| 10 | 131.1 |  |  | 10′ | 144.6 |  |
| 11 | 35.5 | 3.82 (d, 10.4) |  | 1′′ | 36.8 | 3.78 (m) |
| 12 | 53.6 | 2.76 (d, 10.4) |  | 2′′ | 122.4 | 5.94 (d, 9.6) |
| 13 | 87.1 |  |  | 3′′ | 133.9 |  |
| 14 | 21.5 | 1.30 (s) |  | 4′′ | 18.1 | 1.76 (s) |
| 15 | 48.7 | 2.62, (dd, 13.2, 6.4) | 5′′ | 26.3 |  | 1.75 (s) |
|  |  | 2.17 (t, 13.2) | 1OH |  |  | 3.54 (s) |

**Dehydro-α-lapachone (4)**

orange crystalline compound, melting point 148 -149 °C, molecular formula; C_15_H_12_O_3_ (m. wt. 240.08) derived from EI^+^ m/z (240.0).

**Table** **4.** ^1^H NMR (500 MHz), ^13^C NMR (100 MHz) and HMBC correlations of dehydro-α-lapachone in CDCl_3_

| **No.** | **^13^C NMR (δ, ppm)** | **Multiplicity** | **^1^H NMR (δ, ppm) (J in Hz)** | **HMBC Correlation** |
| --- | --- | --- | --- | --- |
| 1 | 181.03 | C | - | - |
| 2 | 118.60 | C | - | - |
| 3 | 152.13 | C | - | - |
| 4 | 180.40 | C | - | - |
| 5 | 126.2 | CH | 8.08 (1H, dd, J = 7.5, 1.5) | C-4, C-7, C-8 |
| 6 | 133.2 | CH | 7.68 (1H, dt, J = 7.5, 1.5) | C-7, C-8 |
| 7 | 133.9 | CH | 7.68 (1H, dt, J = 7.5, 1.5) | C-5, C-6 |
| 8 | 126.2 | CH | 8.08 (1H, dd, J = 7.5, 1.5) | C-1, C-7, C-10 |
| 9 | 135.0 | C | - | - |
| 10 | 134.12 | C | - | - |
| 11 | 115.5 | CH | 6.64 (1H, d, J = 10.0) | C-1, C-3, C-12, C-13 |
| 12 | 130.9 | CH | 5.72 (1H, d, J = 10.0) | C-2, C-3, C-13, C-14, C-15 |
| 13 | 80.1 | C | - | - |
| 14 | 29.4 | CH_3_ | 1.23 (3H, s) | C-11, C-12, C-15 |
| 15 | 28.4 | CH_3_ | 1.53 (3H, s) | C-3, C-11, C-13, C-14 |

**Indanone (Methyl-1,2-dihydroxy-2-(3-methylbut-2-en-1-yl)-3-oxo-2,3-dihydro-1H-indene-1- carboxylate)**

Colourless crystalline compound, m.p.: 220-221°C, (Lit. 220-221 °C) [10]. HR-ESIMS: 290.133 [M+H]^+^ (Calculated for C_16_H_18_O_5_ = 290.12). UV (CHCl_3_) λ_max_ (log ε): 230 (5.27), 240 (5.29) nm. IR ῡ: 3379.2 (O-H, st.), 1724.3, 1725.5 (C=O, carbonyl st.) cm^-1^. ^1^H and ^13^C-NMR:

**Table 5.** 1H NMR and 13C NMR spectroscopic data for indanone **2** in CDCl3

| **C. No.** | **^13^C (δ)** | **^1^H (δ) (*J* in Hz)** | **C. No.** | **^13^C (δ)** |  | **^1^H (δ) (*J* in Hz)** |
| --- | --- | --- | --- | --- | --- | --- |
| 1 | 201.9 |  |  | 10 | 172.8 |  |
| 2 | 83.1 |  |  | 11 | 53.8 | 3.66 (s) |
| 3 | 87.3 |  |  | 1′ | 33.8 | 2.78 (dd, 14.8, 8.4) |
| 4 | 123.4 | 7.80 (dd, 7.6, 1.6) |  |  |  | 2.10 (dd, 14.8, 7.2) |
| 5 | 130.1 | 7.54 (m) |  | 2′ | 116.3 | 5.07 (dd, 8.4, 7.2) |
| 6 | 135.4 | 7.70 (m) |  | 3′ | 137.7 |  |
| 7 | 124.2 | 7.62 (dd, 7.6. 1.6) |  | 4′ | 25.9 | 1.60 (s) |
| 8 | 148.4 |  |  | 5′ | 17.9 | 1.50 (s) |
| 9 | 134.9 |  |  | 2OH | 3.00 (s) | 4.35 (s) |

**Lapachol (1)**

**
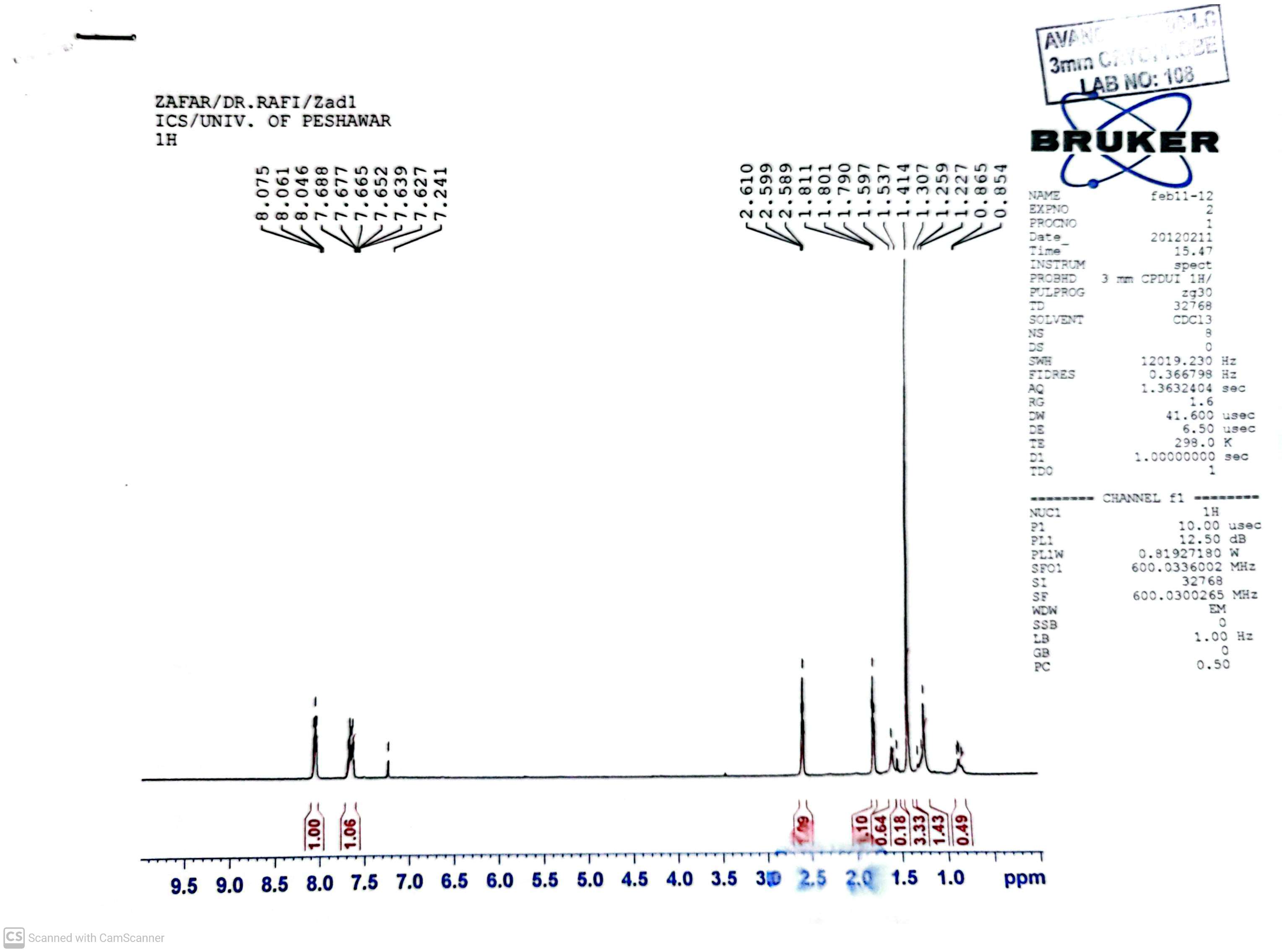
**

Figure S1. The ^1^H-NMR spectrum of compound 1


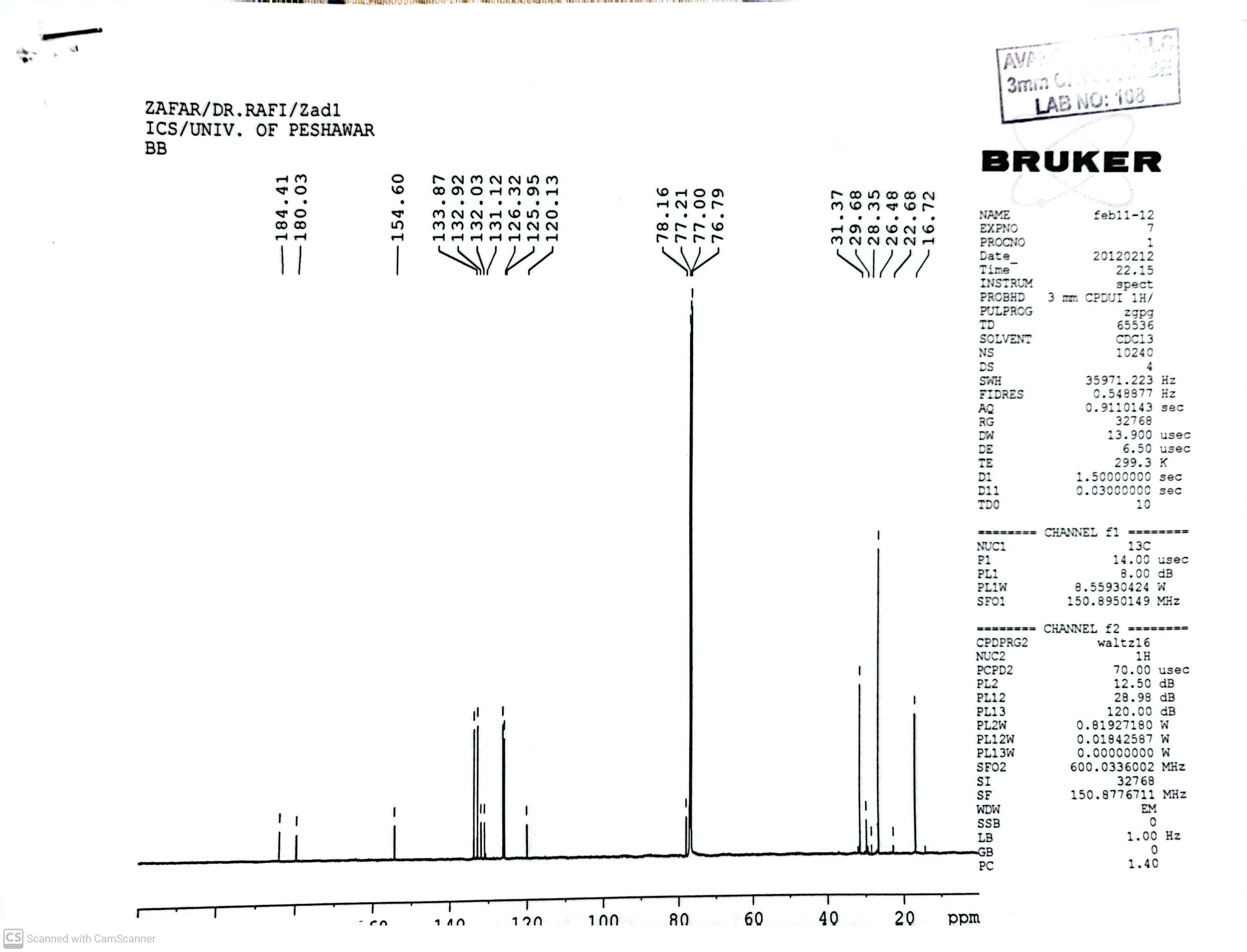


Figure S2. ^13^C-NMR spectrum of compound 1


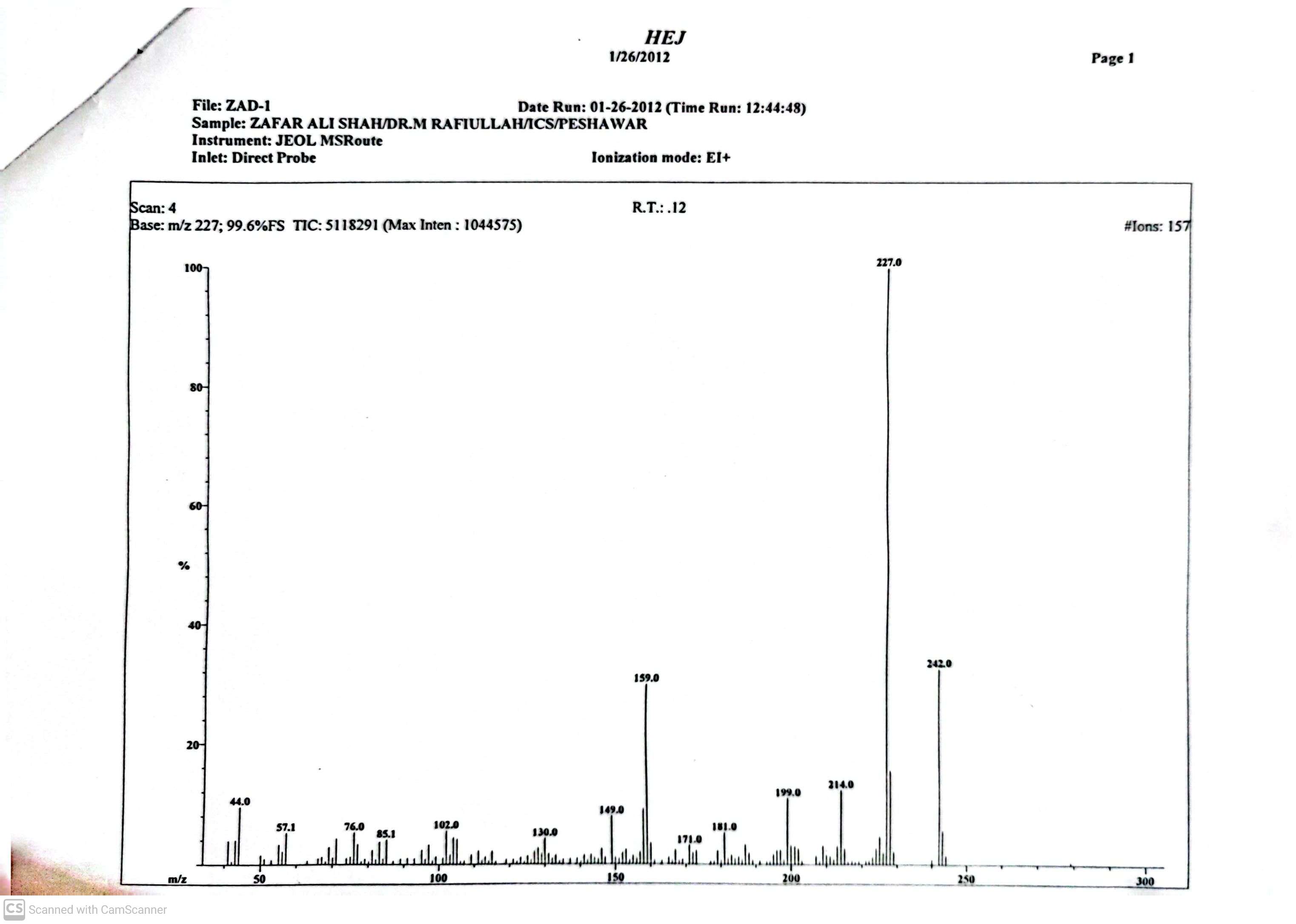


Figure S3: EI MS spectrum of compound 1

**α-Lapachone (2)**

**\**

**Figure 4:** . The ^1^H-NMR spectrum of compound 2

**Figure 5:** ^13^C-NMR spectrum of compound 2

**Figure 6:** EI MS spectrum of compound 2

**Peshawaraquinone (3)**

**Figure 7:** . The ^1^H-NMR spectrum of compound 3

**Figure 8:** ^13^C-NMR spectrum of compound 3

**Figure 9:** EI MS spectrum of compound 3

**Dehydro-α-lapachone (4)**

**
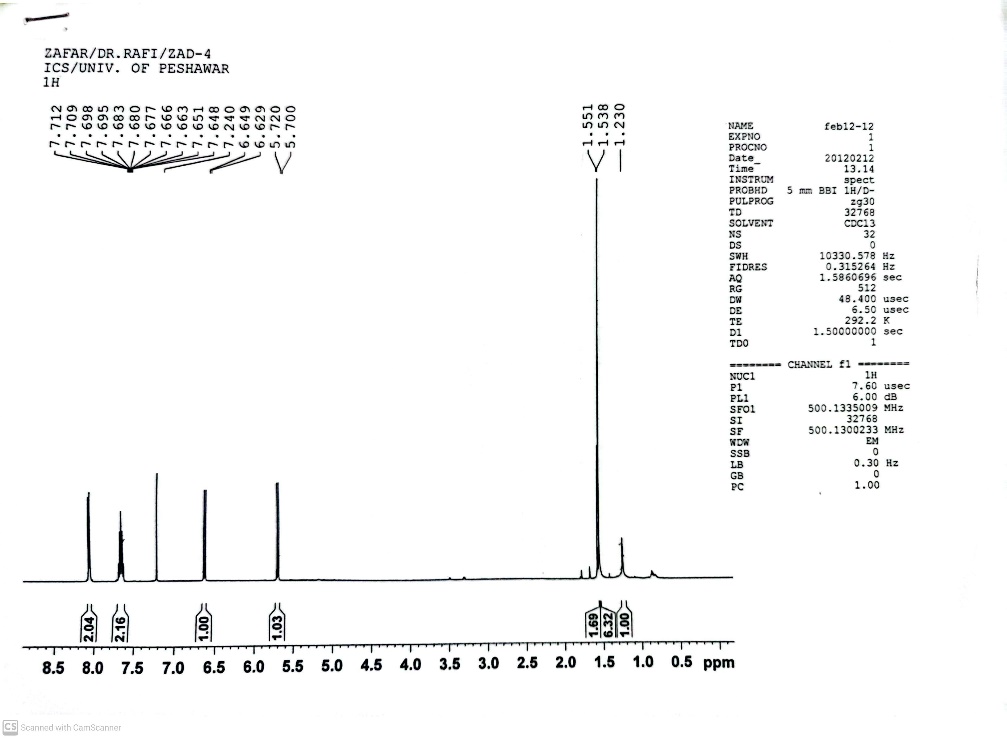
**


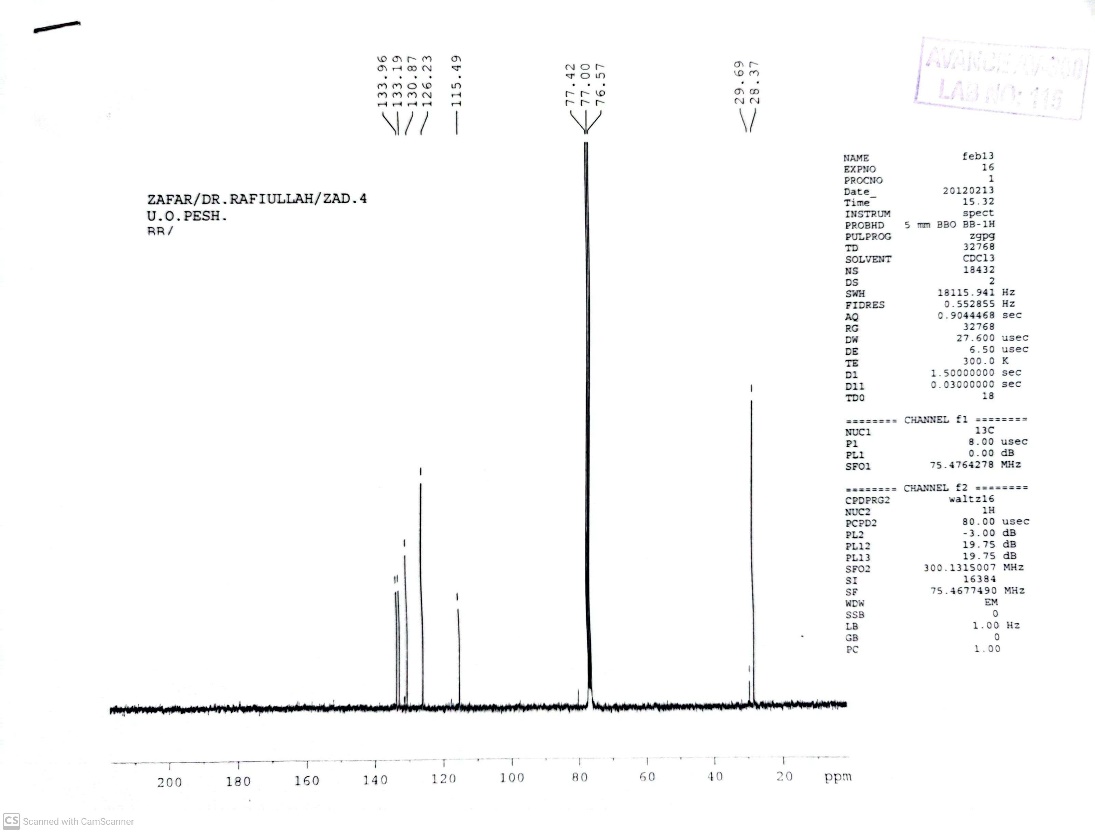
Figure 10: The ^1^H-NMR spectrum of compound 4

Figure 11: ^13^C-NMR spectrum of compound 4

**
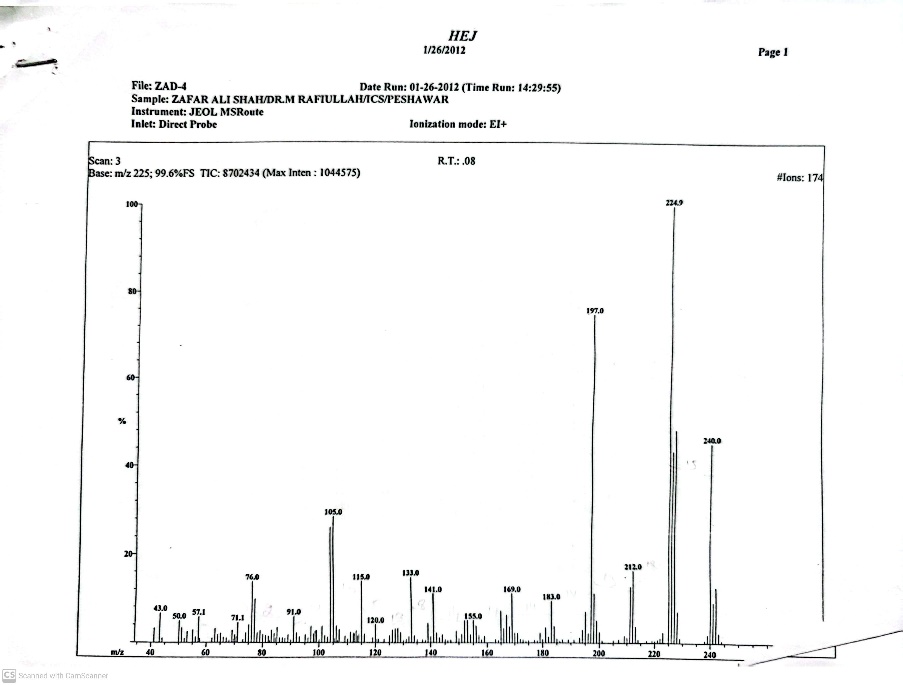
**

**Figure 12:** EI MS spectrum of compound 4

**Indanone**

**Figure 13:** The ^1^H-NMR spectrum of compound 5

**Figure 4:**

**Figure 14:** ^13^C-NMR spectrum of compound 5

**Figure 15:** EI MS spectrum of compound 5

**IC_50_ graphs for bioactive compounds**


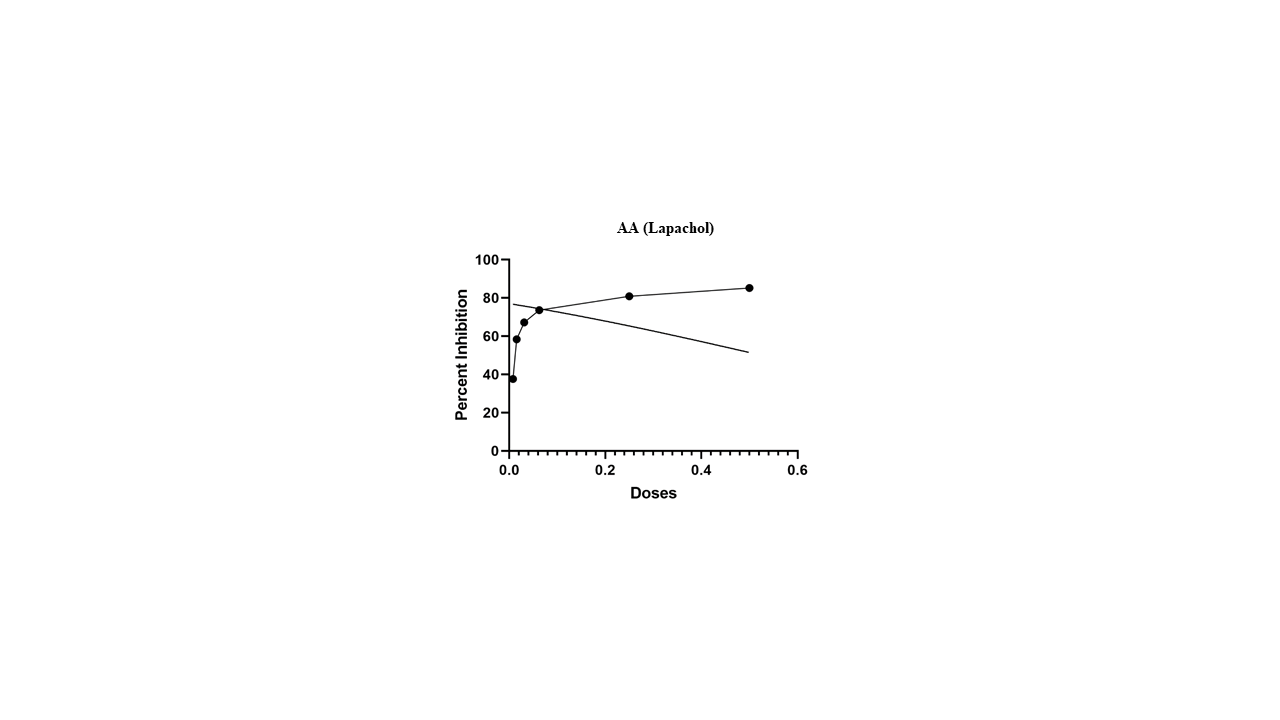


**Figure 16:** IC_50_ graphs for AA: Lapachol


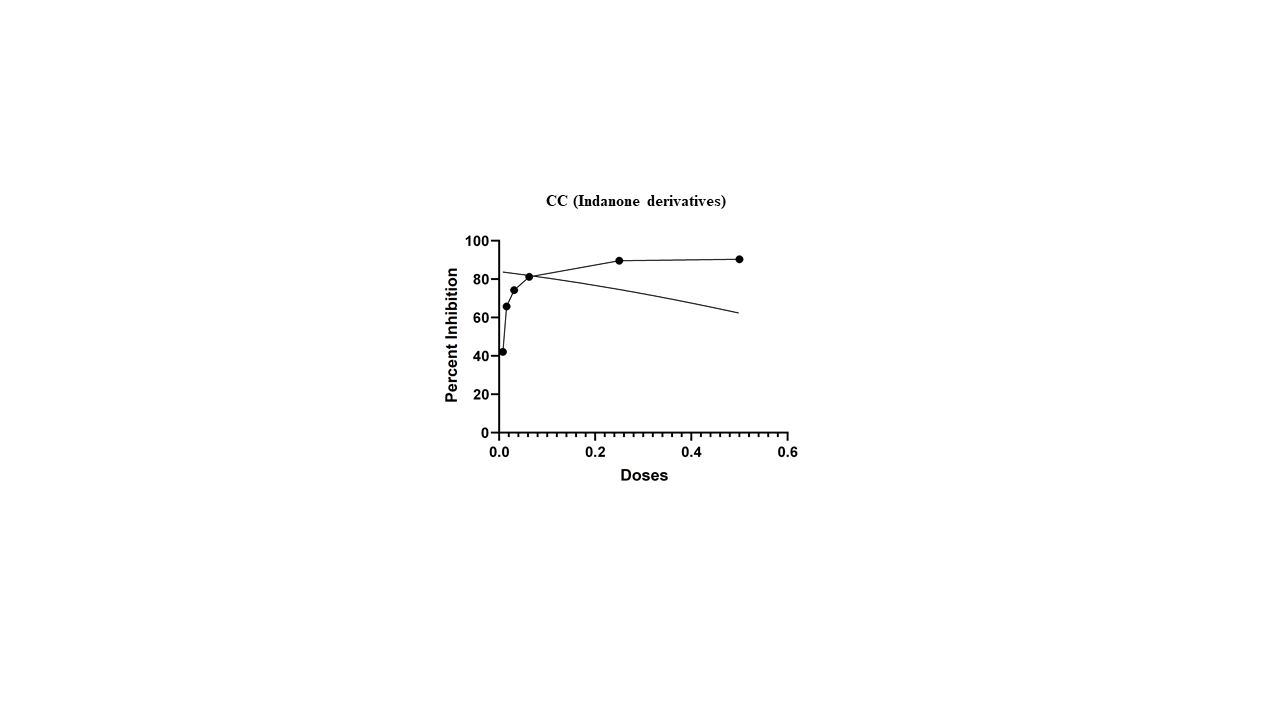


**Figure 17:** IC_50_ graphs for DD: Alpha-lapachone


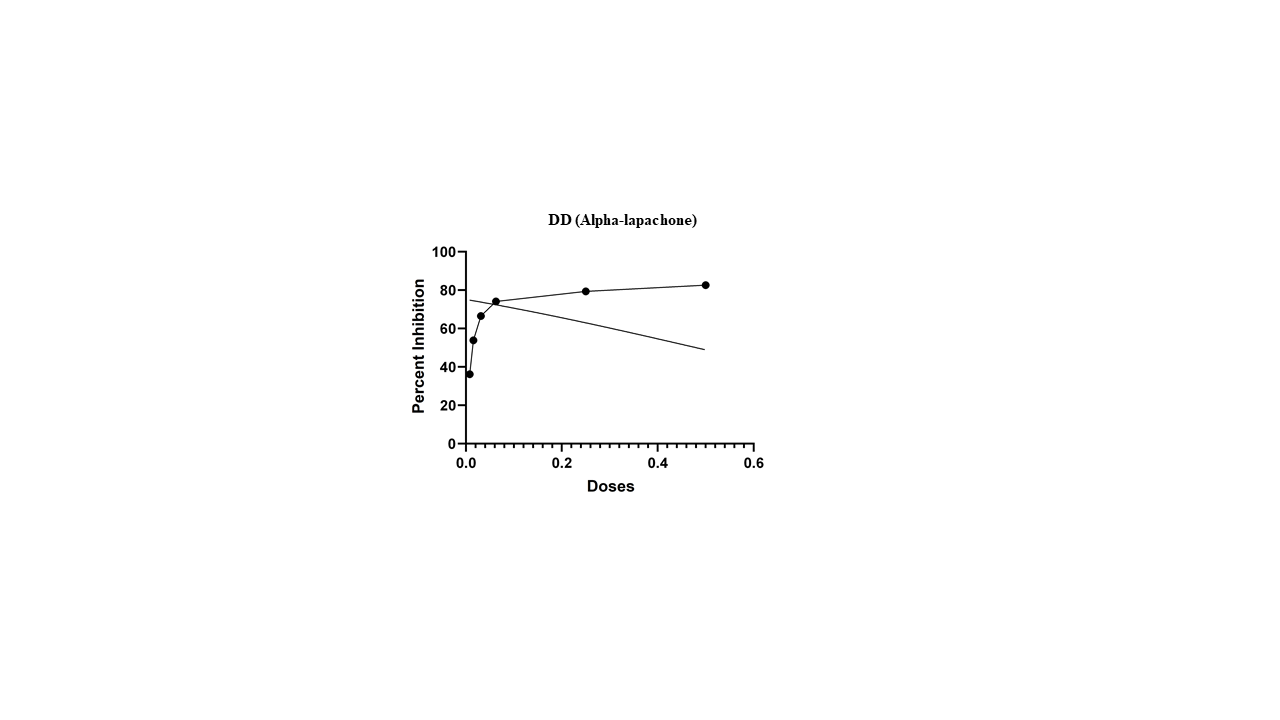


**Figure 18:** IC_50_ graphs for CC: Indanone derivatives


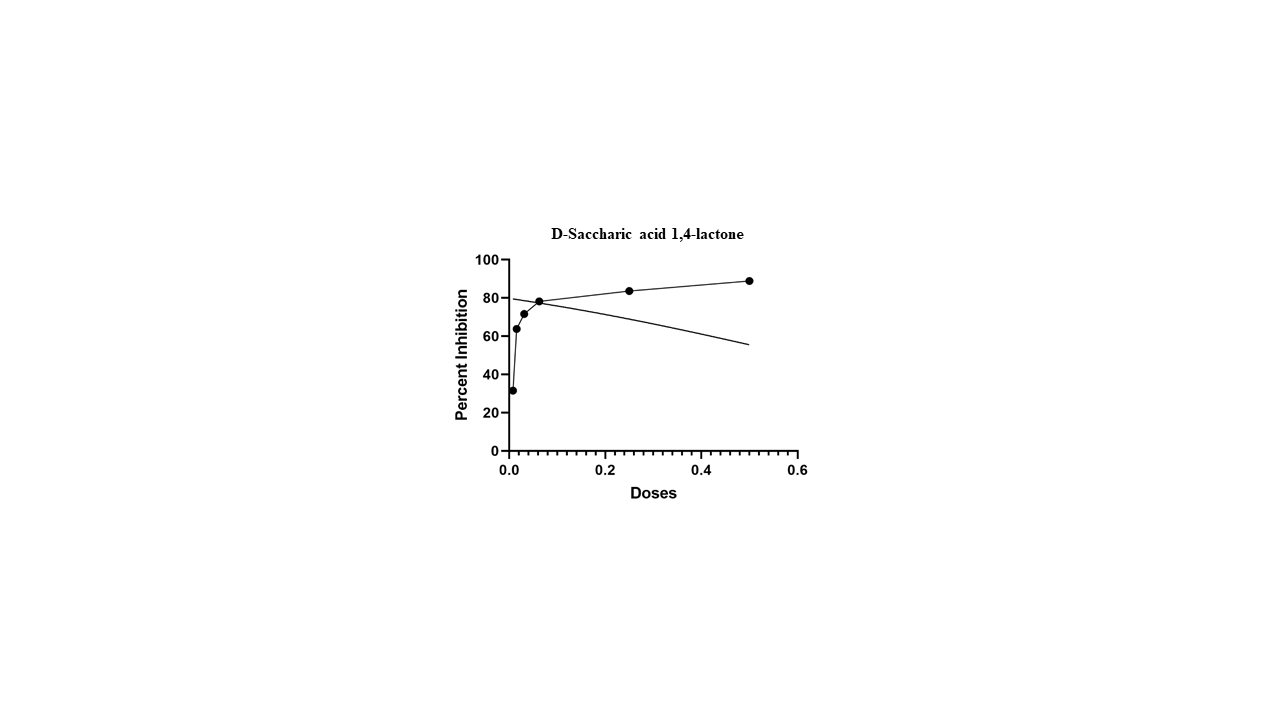


**Figure 19:** IC50 graphs for D-Saccharic acid 1,4-lactone
