# Supplementary material for: Investigation of the inhibitory potential of secondary metabolites isolated from Fernandoa adenophylla against Beta-glucuronidase via molecular docking and molecular dynamics simulation studies
Source: PLoS One. 2025 May 23;20(5):e0324100. doi: 10.1371/journal.pone.0324100 (PMC12101693; doi:10.1371/journal.pone.0324100)
Supplement: Attachment — Submitted filename: comments.pdf [file pone.0324100.s002.pdf]

In this study: " **Investigation of the inhibitory potential of secondary metabolites isolated from *Fernandoa adenophylla* against Beta-glucuronidase *via* molecular docking and molecular dynamics simulation studies**" The authors provide report about the inhibitory activities of compounds isolated from *Fernandoa adenophylla* against β-glucuronidase via in vitro and in silico studies. However, there are many concerns in this study, and major revision is required:

1. Abstract: Avoid non-standard or uncommon abbreviations. If any are essential to include, ensure they are defined within your abstract at first mention.

2. Section 2.1: - Details on chromatographic separation techniques are missing (type of chromatographic column, stationary/mobile phase used, elution conditions, detection).

- Details of spectroscopic techniques to determine the structure of the isolated compound (type of NMR spectroscopy, mass, equipment used).

3. Section 2.3: check again the writing here. There are many grammar and presentation issues. Additionally, all **NMR data** along with **Chemical properties and spectra** are mandatory in Supplemental materials.

4. Section 2.5: references of enzyme structures downloaded from Protein Data Bank are required. In addition, was a grid box defined around the binding site to specify the docking region, or was blind docking used in this study?

Please clearly indicate the grid box parameters (grid size, grid position) for each target protein.

How was the 3D structure of the inhibitor constructed? Was the structure of the test compounds minimized before the docking process? Please indicate.

5. Section 3.1: Please provide the IC_{50} graphs of all test compounds.

The β-glucuronidase enzyme has multiple ligand binding sites (e.g., catalytic and allosteric sites). Without conducting enzyme kinetics analysis, it is unclear which binding site is relevant for the inhibitors. Therefore, to validate the binding site and confirm the docking results, **enzyme kinetics experiments are mandatory**.

6. Section 3.2 Molecular docking analysis: There is much literature reporting the inhibition mode and binding site of β-glucuronidase inhibitors. Please compare your compounds with reported inhibitors. What is the difference between the binding sites? Please clearly describe.

7. Section 3.3 MD simulation: A molecular dynamics simulation of 20 ns is not meaningful and does not yield any significant scientific results. In fact, the protein-ligand complex tends to exhibit many fluctuations during the initial 20 ns equilibration of simulations. Therefore, a prolonged **molecular dynamics simulation of at least 100 ns is required**.

8. It would be beneficial to report the RMSD of the ligand after fitting to the protein backbone, along with the distance between the centers of mass of the protein and ligand. These parameters can reveal any potential internal motions, particularly interdomain motion, within the protein.

9. How about the binding affinity of bound ligand conformations? MM/PB(GB)SA calculations are required.
