# Supplementary material for: Investigation of the inhibitory potential of secondary metabolites isolated from Fernandoa adenophylla against Beta-glucuronidase via molecular docking and molecular dynamics simulation studies
Source: PLoS One. 2025 May 23;20(5):e0324100. doi: 10.1371/journal.pone.0324100 (PMC12101693; doi:10.1371/journal.pone.0324100)
Supplement: Attachment — Submitted filename: Reply-comments.docx [file pone.0324100.s004.docx]

Dear Viet Phong Nguyen, Ph.D.
Academic Editor
PLOS ONE

Thank you very much for the reviewers’ comments concerning our manuscript. We have studied the reviewer comments carefully and have made several revisions to the text. We would like to express our appreciation to you and the reviewers for your many suggestions, which have greatly improved our manuscript. All changes are shown in yellow, highlighted in the revised manuscript, and are outlined below on a point-by-point basis (red color).

We hope these corrections and revisions are satisfactory and that the manuscript now meets the requirements for publication.

We look forward to hearing from you at your earliest convenience.

**Journal Requirements:**

When submitting your revision, we need you to address these additional requirements.

1. Please ensure that your manuscript meets PLOS ONE's style requirements, including those for file naming. The PLOS ONE style templates can be found at https://journals.plos.org/plosone/s/file?id=wjVg/PLOSOne_formatting_sample_main_body.pdf and 
https://journals.plos.org/plosone/s/file?id=ba62/PLOSOne_formatting_sample_title_authors_affiliations.pdf

**Response:** Done

2. Please note that PLOS ONE has specific guidelines on code sharing for submissions in which author-generated code underpins the findings in the manuscript. In these cases, we expect all author-generated code to be made available without restrictions upon publication of the work. Please review our guidelines at https://journals.plos.org/plosone/s/materials-and-software-sharing#loc-sharing-code and ensure that your code is shared in a way that follows best practice and facilitates reproducibility and reuse.

**Response:** Done

3. Please provide a complete Data Availability Statement in the submission form, ensuring you include all necessary access information or a reason for why you are unable to make your data freely accessible. If your research concerns only data provided within your submission, please write "All data are in the manuscript and/or supporting information files" as your Data Availability Statement.

**Response:** Done

**Additional Editor Comments :**

1. Abstract: Avoid non-standard or uncommon abbreviations. If any are essential to include, ensure they are defined within your abstract at first mention.

**Response:** Corrected as suggested.
2. Section 2.1: - Details on chromatographic separation techniques are missing (type of chromatographic column, stationary/mobile phase used, elution conditions, detection).

**Response:** Details on chromatographic separation techniques, including column specifications, stationary and mobile phases, elution conditions, and detection methods, have now been incorporated.

- Details of spectroscopic techniques to determine the structure of the isolated compound (type of NMR spectroscopy, mass, equipment used).

**Response:** Details of the spectroscopic data, along with the corresponding spectra, are provided in the supplementary file.
3. Section 2.3: check again the writing here. There are many grammar and presentation issues.
Additionally, all NMR data along with Chemical properties and spectra are mandatory in Supplemental materials.

**Response:** All grammatical issues have been addressed. The NMR data, physical data, and spectra are now provided in the Supplemental Materials.

Section 2.5: references of enzyme structures downloaded from Protein Data Bank are required. In addition, was a grid box defined around the binding site to specify the docking region, or was blind docking used in this study?

Response: Dear Reviewer, Thank you for your valuable comments. We have incorporated the suggested references and followed the docking procedure as reported in a previous study. (<https://doi.org/10.3390/pr11030880>, doi:10.5281/zenodo.3576583).

Please clearly indicate the grid box parameters (grid size, grid position) for each target protein. How was the 3D structure of the inhibitor constructed? Was the structure of the test compounds minimized before the docking process? Please indicate.

**Response:** The requested details have now been incorporated into the revised manuscript and have been highlighted for clarity.

Section 3.1: Please provide the IC50 graphs of all test compounds.

**Response:** We sincerely appreciate the reviewer's insightful comment. In response, we have incorporated the IC₅₀ graphs for all tested compounds (AA: Lapachol, DD: Alpha-lapachone, CC: Indanone derivatives, and D-Saccharic acid 1,4-lactone) as requested. The IC₅₀ values were determined using nonlinear regression analysis in GraphPad Prism, and the corresponding dose-response curves are now provided below.

The β-glucuronidase enzyme has multiple ligand binding sites (e.g., catalytic and allosteric sites). Without conducting enzyme kinetics analysis, it is unclear which binding site is relevant for the inhibitors. Therefore, to validate the binding site and confirm the docking results, enzyme kinetics experiments are mandatory.

**Response:** Thank you for your valuable comment. We acknowledge that β-glucuronidase has multiple ligand-binding sites, including catalytic and allosteric sites, and that enzyme kinetics experiments are essential for definitive validation. In our study, molecular docking was employed as an initial approach to predict potential interactions. However, to further substantiate our findings, we plan to conduct enzyme kinetics experiments in future studies to confirm the binding site and validate the docking results experimentally.

Section 3.2 Molecular docking analysis: There is much literature reporting the inhibition mode and binding site of β-glucuronidase inhibitors. Please compare your compounds with reported inhibitors. What is the difference between the binding sites? Please clearly describe.

**Response:** The compounds have now been compared with the reported inhibitors, and the updates are highlighted in the revised manuscript.

Section 3.3 MD simulation: A molecular dynamics simulation of 20 ns is not meaningful and does not yield any significant scientific results. In fact, the protein-ligand complex tends to exhibit many fluctuations during the initial 20 ns equilibration of simulations. Therefore, a prolonged molecular dynamics simulation of at least 100 ns is required.

**Response:** The MD simulation has been extended to 100 ns, and the updates have been highlighted in the revised manuscript.

It would be beneficial to report the RMSD of the ligand after fitting to the protein backbone, along with the distance between the centers of mass of the protein and ligand. These parameters can reveal any potential internal motions, particularly interdomain motion, within the protein.

**Response:** As per the suggestions, the RMSD and distance analysis have been incorporated and highlighted in the revised manuscript.

How about the binding affinity of bound ligand conformations? MM/PB(GB)SA calculations are required

**Response:** The required details have now been added and appropriately highlighted in the revised manuscript.
