# Supplementary material for: Investigation of the inhibitory potential of secondary metabolites isolated from Fernandoa adenophylla against Beta-glucuronidase via molecular docking and molecular dynamics simulation studies
Source: PLoS One. 2025 May 23;20(5):e0324100. doi: 10.1371/journal.pone.0324100 (PMC12101693; doi:10.1371/journal.pone.0324100)
Supplement: Attachment — Submitted filename: Comments reply.docx [file pone.0324100.s005.docx]

Dear Viet Phong Nguyen, Ph.D.
Academic Editor
PLOS ONE

Thank you very much for the reviewers’ comments concerning our manuscript. We have carefully reviewed the comments from the reviewers and made several revisions to the text. We would like to express our appreciation to you and the reviewers for your many suggestions, which have greatly improved our manuscript. All changes are highlighted in yellow in the revised manuscript and are outlined below on a point-by-point basis (in red).

We hope these corrections and revisions are satisfactory and that the manuscript now meets the requirements for publication.

We look forward to hearing from you at your earliest convenience.

**Journal Requirements:**

Please review your reference list to ensure that it is complete and correct. If you have cited papers that have been retracted, please include the rationale for doing so in the manuscript text, or remove these references and replace them with relevant current references. Any changes to the reference list should be mentioned in the rebuttal letter that accompanies your revised manuscript. If you need to cite a retracted article, indicate the article’s retracted status in the References list and also include a citation and full reference for the retraction notice.

**Response:** The reference list has been checked and found to be corrected.

**Reviewers' comments:**

Reviewer's Responses to Questions

**Comments to the Author**

1. If the authors have adequately addressed your comments raised in a previous round of review and you feel that this manuscript is now acceptable for publication, you may indicate that here to bypass the “Comments to the Author” section, enter your conflict of interest statement in the “Confidential to Editor” section, and submit your "Accept" recommendation.

Reviewer #1: All comments have been addressed

Reviewer #2: All comments have been addressed

Reviewer #3: All comments have been addressed

**Response:** Thank you.

2. Is the manuscript technically sound, and do the data support the conclusions?

The manuscript must describe a technically sound piece of scientific research with data that supports the conclusions. Experiments must have been conducted rigorously, with appropriate controls, replication, and sample sizes. The conclusions must be drawn appropriately based on the data presented.

Reviewer #1: Yes

Reviewer #2: Partly

Reviewer #3: Yes

**Response:** The necessary corrections have been made.

3. Has the statistical analysis been performed appropriately and rigorously?

Reviewer #1: N/A

Reviewer #2: Yes

Reviewer #3: (No Response)

**Response:** Thank you.

4. Have the authors made all data underlying the findings in their manuscript fully available?

The [PLOS Data policy](http://track.editorialmanager.com/CL0/http:%2F%2Fwww.plosone.org%2Fstatic%2Fpolicies.action%23sharing/1/010f01961be2d3ac-794797b5-af67-4dc6-857a-1b76a01cbb1c-000000/ULUNexWO0QokMSMGKXlOuKc3oOkbddBbwfqQEbHi2UA=206) requires authors to make all data underlying the findings described in their manuscript fully available without restriction, with rare exception (please refer to the Data Availability Statement in the manuscript PDF file). The data should be provided as part of the manuscript or its supporting information, or deposited to a public repository. For example, in addition to summary statistics, the data points behind means, medians and variance measures should be available. If there are restrictions on publicly sharing data—e.g. participant privacy or use of data from a third party—those must be specified.

Reviewer #1: Yes

Reviewer #2: Yes

Reviewer #3: (No Response)

**Response:** Thank you.

5. Is the manuscript presented in an intelligible fashion and written in standard English?

PLOS ONE does not copyedit accepted manuscripts, so the language in submitted articles must be clear, correct, and unambiguous. Any typographical or grammatical errors should be corrected at revision, so please note any specific errors here.

Reviewer #1: Yes

Reviewer #2: Yes

Reviewer #3: (No Response)

**Response:** Thank you.

**6. Review Comments to the Author**

Please use the space provided to explain your answers to the questions above. You may also include additional comments for the author, including concerns about dual publication, research ethics, or publication ethics. (Please upload your review as an attachment if it exceeds 20,000 characters)

Reviewer #1: The authors adequately responded to all comments and suggestions raised to improve their manuscript.

**Response:** Thank you.

Reviewer #2: In my opinion, the manuscript “Investigation of the inhibitory potential of secondary metabolites isolated from Fernandoa adenophylla against Beta-glucuronidase via molecular docking and molecular dynamics simulation studies” is well written and presents interesting information. But I would like to point out some issues which can be revised:
Abstract: Revise the abstract to align with conventional standards of clarity, logical flow, and objectivity.

**Response:** Dear reviewer, thank you for your suggestion. We have revised the abstract to make it more concise and clear.

Introduction: The introduction section should be rewritten in a more focused manner, in concise paragraphs. The statement of the problem and the rationale of this study is unclear. Although the authors have included some studies, it is needed to include additional literature on this plant, and a justification of its choice for the current investigation. According to this study, isolated chemical constituents (1-5) of Fernandoa adenophylla exhibited remarkable antidiabetic activity. Additionally, what are all the major phytocompounds have been reported and so on can be added.

**Response:** Dear reviewer, thank you for your suggestion. The introduction has been revised as per the suggestions.

Result: Appropriate inferential statistics are missing, especially for results. This is particularly important in interpreting the results, appraising their validity, and drawing appropriate conclusions.

**Response:** We thank the reviewer for the comment. The results were presented as mean ± SEM from triplicate experiments, analyzed using ANOVA to ensure consistency. Due to the limited sample size (n=3), further inferential statistics (e.g., p-values) were not applied to avoid overinterpretation. However, conclusions were supported through complementary molecular docking, MD simulations, and MM-GBSA analyses, providing robust validation of the findings.

Discussion : The discussion section should be revised and focused on interpretation, relationships with reports from other scholars in the field, and implication of the obtained results.

**Response:** Dear reviewer, we have revised the discussion as per the suggestions

Reviewer #3: I recommend the acceptance of the manuscript. One additional comment I just mention that author should write the unit of binding energy in the manuscript.

**Response:** We thank the reviewer for the suggestion. The unit of binding energy (kcal/mol) has been added in the text and Table 4.

7. PLOS authors have the option to publish the peer review history of their article ([what does this mean?](https://track.editorialmanager.com/CL0/https:%2F%2Fjournals.plos.org%2Fplosone%2Fs%2Feditorial-and-peer-review-process%23loc-peer-review-history/1/010f01961be2d3ac-794797b5-af67-4dc6-857a-1b76a01cbb1c-000000/i-G71ts7K2ww2zMQmJBvUi7VLFxQQ0sOIQMOflwfO8s=206)). If published, this will include your full peer review and any attached files.


If you choose “no”, your identity will remain anonymous but your review may still be made public.


**Do you want your identity to be public for this peer review?** For information about this choice, including consent withdrawal, please see our [Privacy Policy](https://track.editorialmanager.com/CL0/https:%2F%2Fwww.plos.org%2Fprivacy-policy/1/010f01961be2d3ac-794797b5-af67-4dc6-857a-1b76a01cbb1c-000000/apne0cqIttCh0U7m9p9NNViObX4BfSs62SXsjrR6AZ0=206).

Reviewer #1: **Yes:** Khaled M. Darwish

Reviewer #2: No

Reviewer #3: **Yes:** Shashank Shekher Mishra

**Response:** It okay
